# Neuregulin 1 confers neuroprotection in SOD1-linked amyotrophic lateral sclerosis mice via restoration of C-boutons of spinal motor neurons

**DOI:** 10.1186/s40478-016-0286-7

**Published:** 2016-02-18

**Authors:** Jurate Lasiene, Okiru Komine, Noriko Fujimori-Tonou, Berit Powers, Fumito Endo, Seiji Watanabe, Jin Shijie, John Ravits, Philip Horner, Hidemi Misawa, Koji Yamanaka

**Affiliations:** Laboratory for Motor Neuron Disease, RIKEN Brain Science Institute, Wako, Saitama 3510198 Japan; Department of Neuroscience and Pathobiology, Research Institute of Environmental Medicine, Nagoya University, Nagoya, Aichi 4648601 Japan; Laboratory for Molecular Dynamics of Mental Disorders, RIKEN Brain Science Institute, Wako, Saitama 3510198 Japan; Department of Neurological Surgery, University of Washington, Seattle, WA 98104 USA; Ionis Pharmaceuticals, Inc., Carlsbad, CA 92010 USA; Department of Neurosciences, University of California, San Diego, La Jolla, CA 92093 USA; Division of Pharmacology, Faculty of Pharmacy, Keio University, Tokyo, 1058512 Japan

**Keywords:** Amyotrophic lateral sclerosis, Neuregulin 1, C-bouton, SOD1, ErbB4

## Abstract

**Introduction:**

Increasing evidence implicates the role of the cell types surrounding motor neurons, such as interneurons and glial cells, in non-cell autonomous neurodegeneration of amyotrophic lateral sclerosis (ALS). C-boutons, the large cholinergic synapses that innervate spinal α-motor neurons to control their excitability, are progressively lost from motor neurons in both human ALS and mutant Cu/Zn superoxide dismutase 1 (SOD1)-ALS mice. Neuregulin-1 (NRG1), a trophic factor implicated in neural development, transmission, and synaptic plasticity, has been reported to localize in the synapse of C-boutons. However, the roles of NRG1 in maintenance of motor neuron health and activity, as well as the functional consequences of its alteration in motor neuron disease, are not fully understood.

**Results:**

NRG1 was localized to the post-synaptic face of C-boutons and its expression was significantly lost in SOD1-ALS mice and human ALS patients. Losses of NRG1 expression and C-boutons occured almost contemporaneously in SOD1-ALS mice. In addition, expressions of ErbB3 and ErbB4, receptors for NRG1, were reduced in the motor neurons of SOD1-ALS mice. Furthermore, viral-mediated delivery of type III-NRG1 to the spinal cord restored the number of C-boutons and extended the survival time of SOD1-ALS mice.

**Conclusions:**

These results suggest that maintenance of NRG1-ErbB4/3 axis by supplementation of NRG1 confers neuroprotection in motor neuron disease, partly through the maintenance of C-boutons of spinal motor neurons.

**Electronic supplementary material:**

The online version of this article (doi:10.1186/s40478-016-0286-7) contains supplementary material, which is available to authorized users.

## Introduction

A key characteristic of neurodegenerative diseases is the degeneration of a specific type of neuron. In amyotrophic lateral sclerosis (ALS), an adult-onset neurodegenerative disease, motor neurons are specifically affected during the degenerative process. While the majority of ALS cases are of sporadic etiology, approximately 10 % of cases are inherited, and dominant mutations in the gene for copper/zinc superoxide dismutase (SOD1) are a frequent cause of inherited ALS. Using transgenic rodent and fish models of SOD1-linked ALS that recapitulate specific motor neuron degeneration, the cell types surrounding motor neurons, including glial cells and interneurons, have been shown to be actively involved in ALS pathogenesis [1–4].

C-boutons are large cholinergic terminals that synapse onto spinal α-motor neurons, which are highly vulnerable in ALS. The origin of C-boutons was recently identified as partition cells, cholinergic interneurons located vicinity of the spinal central canal, and their role uncovered as critical modulators of motor neuron activity during locomotor behavior [5–7]. Intriguingly, C-boutons do not terminate on the cranial motor neurons innervating eye muscles, which are spared in ALS. In sporadic ALS patients, losses of C-boutons on spinal motor neurons remaining at autopsy were documented [8]. However, alteration of C-boutons in symptomatic mutant SOD1 mice reported by several groups was variable: one report showed a significant loss of C-boutons [9], while the other groups reported moderate [10] or marginal [11, 12] loss of C-boutons. Moreover, the reports demonstrate early reduction of choline acetyltransferase (ChAT) at C-boutons in presymptomatic SOD1^G93A^ mice [13] and reduced immunoreactivity for vesicular acetylcholine transporter (VAChT) in motor neurons in 80 day-old, presymptomatic SOD1^G93A^ mice [14]. Others demonstrate a decrease in VAChT-positive boutons following symptomatic onset in mouse models [9, 15]. These reports suggest that C-boutons could be linked to pathomechanism of ALS, however, existing evidence of altered numbers and roles of C-boutons in ALS patients and models is contradictory and largely inconclusive.

Neuregulin 1 (NRG1), is an epidermal growth factor (EGF)-like trophic factor implicated in neural development, synaptic transmission and plasticity, Schwann cell differentiation, and the regulation of myelin sheath thickness [16, 17]. NRG1 is localized to the cholinergic synapses (C-boutons) that innervate motor neuron somata and proximal processes [18] and modulate the activity of motor neurons. In addition to known involvement of NRG1 in schizophrenia [17], recent data suggests the possible involvement of NRG1 in ALS. A dominant mutation in the gene for ErbB4, encoding a receptor for NRG1, is causative for familial ALS type 19 [19]. Moreover, although the results are not entirely consistent with each other, altered expression of NRG1 has been reported in the lesions of mutant SOD1 mice [9, 20]. These accumulating data suggest that NRG1 could participate in stabilizing synapses or in regulating activity of motor neurons at C-boutons and that its dysregulation could contribute to motor neuron degeneration in ALS. However, its localization in normal and diseased motor neurons and whether NRG1 has neuroprotective properties are not fully understood.

In this study, we found that NRG1 was localized in the post-synaptic face of C-boutons and its expression was significantly lost in SOD1-linked ALS mice and human ALS patients. Viral-mediated delivery of type III NRG1 restored the number of C-boutons and extended the survival time of SOD1^G93A^ mice. These results suggest that NRG1 exerts neuroprotective properties in motor neuron disease partly through maintenance of C-boutons of spinal motor neurons.

## Materials and Methods

### Transgenic mouse lines and survival experiments

Transgenic mouse lines expressing human SOD1 gene with ALS-linked mutations, SOD1^G93A^ [B6.Cg-Tg(SOD1*G93A)1Gur/J] or SOD1^G85R^ [B6.Cg-Tg(SOD1*G85R)148Dwc/J] were obtained from Jackson laboratory or were gifts from Dr. Don Cleveland (University of California, San Diego). Onset and survival times for mutant SOD1 mice are 100–105 and 150–160 days old for SOD1^G93A^ mice, and 11–12 and 12–13 months old for SOD1^G85R^ mice, respectively. Genotyping of SOD1 transgenic mice was identified by polymerase chain reaction (PCR) as previously described [21].

All animal procedures were conducted in accordance with the guidelines of the Animal Use and Care Committees of Nagoya University, Keio University and RIKEN. Transgenic animals are always compared with their non-transgenic littermates to minimize the effects of different genetic background. Times of disease onset and end stage were determined respectively as the time when mice had started losing the maximum body weight and when animals failed in righting themselves within 20 s when placed on their backs, an endpoint commonly used for mutant SOD1 expressing mice which is compliant with the requirements of the Animal Use and Care Committee. Statistical analysis of onset and survival time was performed with Gehan-Breslow-Wilcoxon test by using GraphPad Prism (GraphPad Software, USA).

### Postmortem human tissues

Spinal cord tissues from three patients with sporadic ALS and three control patients who died of causes other than ALS (patient data summarized in Table 1) were obtained by autopsy with informed consent. The diagnosis of ALS was confirmed by El Escorial diagnostic criteria as defined by the World Federation of Neurology. These ALS patients were confirmed to be negative for *C9Orf72* expansion or other known causes of inherited ALS. The collection of tissues and their use in this study was approved by the institutional review board for research ethics of Benaroya Research Institute, Seattle, WA, USA.

**Table 1:** Detail of the patients. The spinal cord specimens from three sporadic ALS and control patients with other diseases were analyzed. Disease controls include Parkinson’s diseases, cerebrovascular disease, and liver failure

ID	Diagnosis		Clinical information				Time of sampling after death (hrs)
Patient ID.	Primary Diagnosis	Secondary Diagnosis	Age at death	Gender	Site of Onset	Disease Course (yrs)	
ALS-43	SALS		74	Male	Respiratory & trunk	1.75	6
ALS-47	SALS	FTD	65	Female	Bulbar	1.25	7
ALS-60	SALS		58	Female	Bulbar	3	3
Control-19	Parkinson's disease		80	Female	NA	NA	2.5
Control-20	Basilar artery occlusion		38	Male	NA	NA	6
Control-44	Liver Failure		80	Female	NA	NA	5

### Antibodies

Following primary antibodies were used in the study: anti-NRG1 (rabbit; 1:500, sc-348, Santa Cruz, USA), anti-VAChT (guinea pig; 1:250, AB1588, Merck Millipore, USA), anti-ChAT (goat; 1:100, AB144P, Merck Millipore), anti-NeuN (mouse; 1:1000, MAB377, Merck Millipore), anti-Kv2.1 (mouse; 1:250, clone K89/34, NeuroMab, USA), anti m2 muscarinic receptor (rat; 1:500, MAB367, Merck Millipore), anti-GFAP (mouse; 1:500, G3893, Sigma-Aldrich, USA), anti-Iba1 (rabbit; 1:500, 019-19741, Wako, Japan), anti-ErbB3 (rabbit; 1:50, sc-285, Santa Cruz), and anti-ErbB4 (mouse; 1:300, MS-270-P0, Thermo Fisher Scientific, USA).

### Immunofluorescence

Immunofluorescence was performed as described previously [21]. For human spinal cords, 20 μm sections were made of fresh frozen tissue, air dried on slides, and fixed in ice-cold acetone for 10 min. In brief, after blocking for 1 h, the sections were incubated with primary antibodies overnight at 4 °C. Bound antibodies were detected with appropriate Alexa Fluor-conjugated anti-rabbit, mouse, rat, goat, or guinea pig IgG antibodies (Thermo Fisher Scientific). For detecting ErbB3 antibody, Cy3-conjugated anti-rabbit IgG antibody (Jackson Laboratories, USA) was used. Immunostained images were obtained by confocal laser scanning microscopy (LSM 5 Exciter, LSM-700; Carl Zeiss AG, Germany) and the equipped software (Zen; Carl Zeiss AG).

### Quantification of NRG1, VAChT-immunopositive puncta and the size of motor neurons

Sections of mouse lumbar spinal cords were triple-immunostained with the antibodies for NRG1, VAChT, and NeuN and appropriate secondaries. After obtaining three-dimensional images of immunostained lumbar spinal cord sections of mice, 30 to 60 motor neurons from two to five animals per genotype were quantified for NRG1- and VAChT- immunoreactivity surrounding motor neurons. Lumbar motor neurons were positively identified as large, NeuN-positive neurons surrounded by VAChT-positive puncta located in the anterior horn. Quantified data were statistically analyzed with one-way ANOVA with Bonferroni’s Multiple Comparison test or Kruskal-Wallis test with Dunn’s Multiple Comparison test.

For human tissue, spinal cord sections were stained with the antibodies for NRG1 and ChAT. After obtaining three-dimensional images of immunostained spinal cord sections, a minimum of 15–20 motor neurons from each of 3 ALS patients at each spinal level (cervical, thoracic, and lumbar) were quantified for NRG1-positive puncta and compared to individuals who died of causes unrelated to ALS. Data was statistically analyzed by one-way ANOVA with Bonferroni correction.

To analyze the size of motor neurons, total of 60 lumbar motor neurons immunopositive for anti-ChAT or anti-NeuN antibody from three mice per each group was quantified for the surface area by using the Image J software.

### Preparation and stereotaxic injection of recombinant adeno-associated virus (AAV) encoding NRG1

The adeno-associated viral expression constructs chimeric rAAV1/2-CAG-NRG1-IRES-EGFP-WPRE (woodchuck post-translational regulatory element) were constructed by subcloning Type I-NRG1 or Type III (SMDF)-NRG1 cDNA into the rAAV1/2 cassette. Viral vectors were packaged and purified at a high titer suitable for expression in mouse spinal cords (GeneDetect, New Zealand). The stereotaxic injections of rAAV into SOD1^G93A^ mice were performed at 65 days old or 105 days old. The mice were anesthetized by intraperitoneal injection of pentobarbital, then 0.5 μl of rAAV was injected bilaterally into the lumbar ventral spinal cord at Th13, L1, and L2 levels of the spine for a total volume of 3 μl, equivalent to 3.6 × 10^8^ viral particles. Similar quantities of rAAV-GFP were also injected into a control group of mice.

### Reverse transcribed PCR

Total RNA was extracted from lumbar spinal cords using TRIzol (Thermo Fisher Scientific) and purified using an RNeasy Mini Kit (Qiagen, Valencia, USA). cDNA was synthesized from total RNA using PrimeScript II 1^st^ strand cDNA Synthesis Kit (Takara Bio, Japan). Quantitative RT-PCR was performed using SYBR Premix Ex Taq II (Takara Bio) using gene specific primers as follows. Type III-NRG1: GAGGTGAGAACACCCAAGTCA, TGGTCCCAGTCGTGGATGTAG; Type I-NRG1: TGCCAACATCACCATTGTTGA, GGACACATAGGGTCTTTCAGTTGA [22]. Primers for Type III-NRG1 were also used for semi-quantitative PCR.

## Results

### Neuregulin 1 is localized to the post-synaptic face of C-bouton in mouse spinal motor neurons

**Fig. 1:**
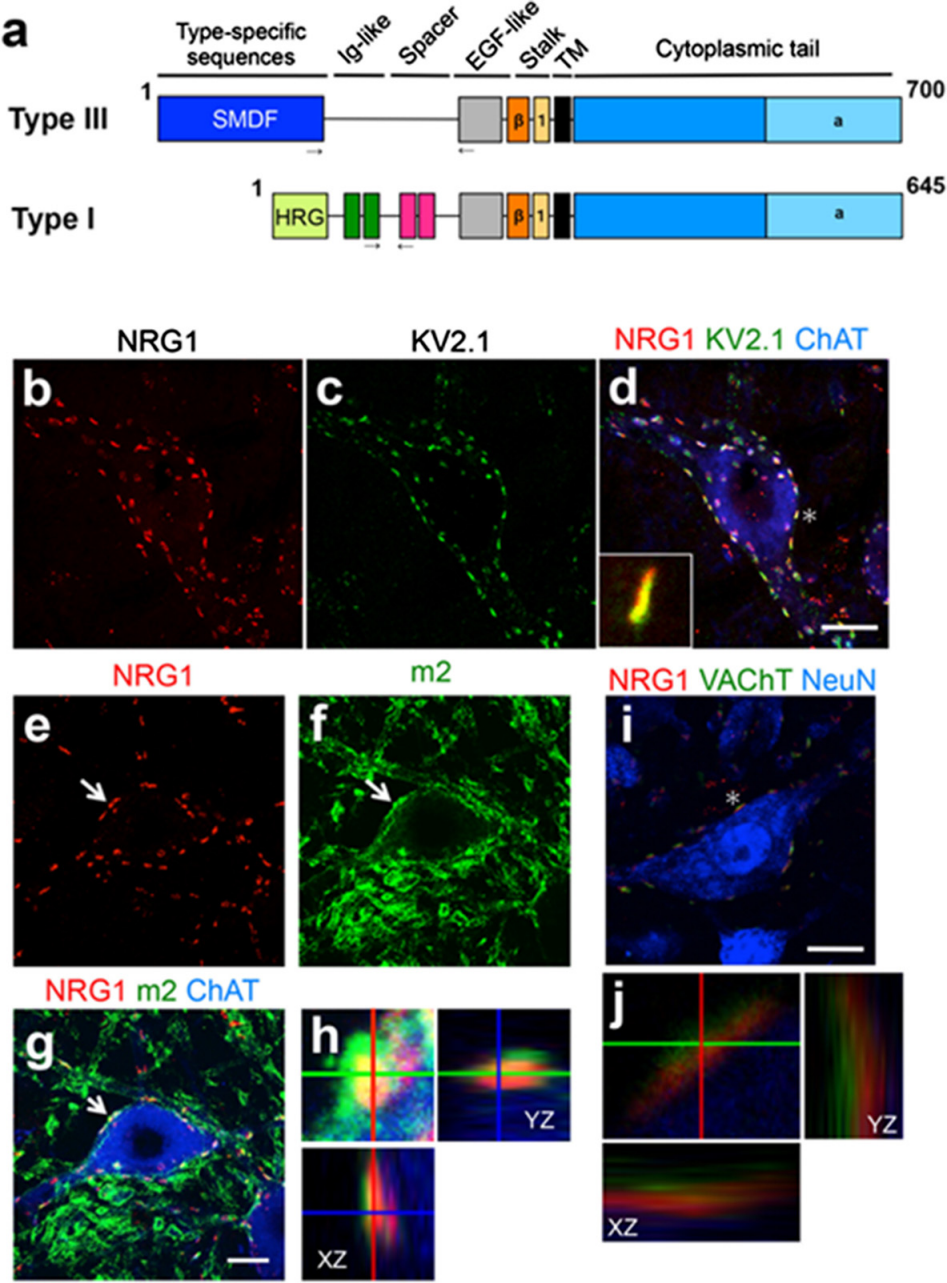
Neuregulin-1 is localized in the post-synaptic face of C-bouton in the mouse spinal motor neurons. (**a**) Domain structures for murine Neuregulin-1 (NRG1) type III (Type III-β1a, based on gene accession number: NM 178591) and type I (Type I-β1a, based on gene accession number: AY648976). The anti-NRG1 antibody used in this study recognizes both Type I and Type III NRG1. *Arrows* indicate the sites of primers used for quantitative PCR in Fig. 2. (**b**–**d**) Representative images of lumbar spinal cord sections from 4 month old wild-type mice (C57BL/6) stained with the antibodies for neuregulin-1 (NRG1) (**b**), Kv2.1 (**c**), along with the merged image stained by NRG-1, Kv2.1, and choline acetyltransferase (ChAT) antibodies (**d**). Inset showed magnified image of C-bouton indicated by asterisk (**d**). (**e**–**h**) Representative images of lumbar spinal cord sections from 4 month old wild-type mice stained with the antibodies for neuregulin-1 (NRG1) (**e**), m2 muscarinic acetylcholine receptor (m2) (**f**), along with the merged image stained with NRG1, m2, and ChAT antibodies (**g**). Magnified Z-axis images of the C-bouton in (**e**–**g**: *arrow*) (**h**). (**i**) Representative image of motor neuron stained by NRG1, VAChT, and NeuN antibodies. (**j**) Magnified Z axis-images of the C-bouton in (**e**: asterisk). Bar: 20 μm (**b**–**d**, **i**), 12.5 μm (**e**–**g**)

To investigate the localization of neuregulin 1 (NRG1) in C-boutons of spinal motor neurons, detailed immunofluorescence analysis was performed. Immunoreactivity for NRG1 showed punctate staining surrounding somata and proximal processes of large motor neurons in mouse lumbar spinal cords (Fig. 1a, b). NRG1 puncta were predominantly co-localized with Kv2.1, a post-synaptic marker for cholinergic synapses on motor neurons (Fig. 1c, d), suggesting the post-synaptic localization of NRG1 on the C-bouton. The post-synaptic localization of NRG1 on the C-bouton was further confirmed by its co-localization with m2 muscarinic acetylcholine receptors on motor neurons (Fig. 1e–h). Vesicular acetylcholine transporter (VAChT) is mainly localized on cholinergic synaptic vesicle membranes and is used as a marker of C-bouton synaptic terminals [18, 23]. Although NRG1 was localized to close vicinity of VAChT-immunoreactivity, NRG1 immunoreactivity did not overlap with VAChT at C-boutons (Fig. 1i, j). These results suggest that NRG1 is specifically localized to the post-synaptic face of C-boutons in mouse spinal motor neurons.

### Age-dependent loss of NRG1 in lumbar spinal motor neurons of SOD1^G93A^ and SOD1^G85R^ mice

**Fig. 2:**
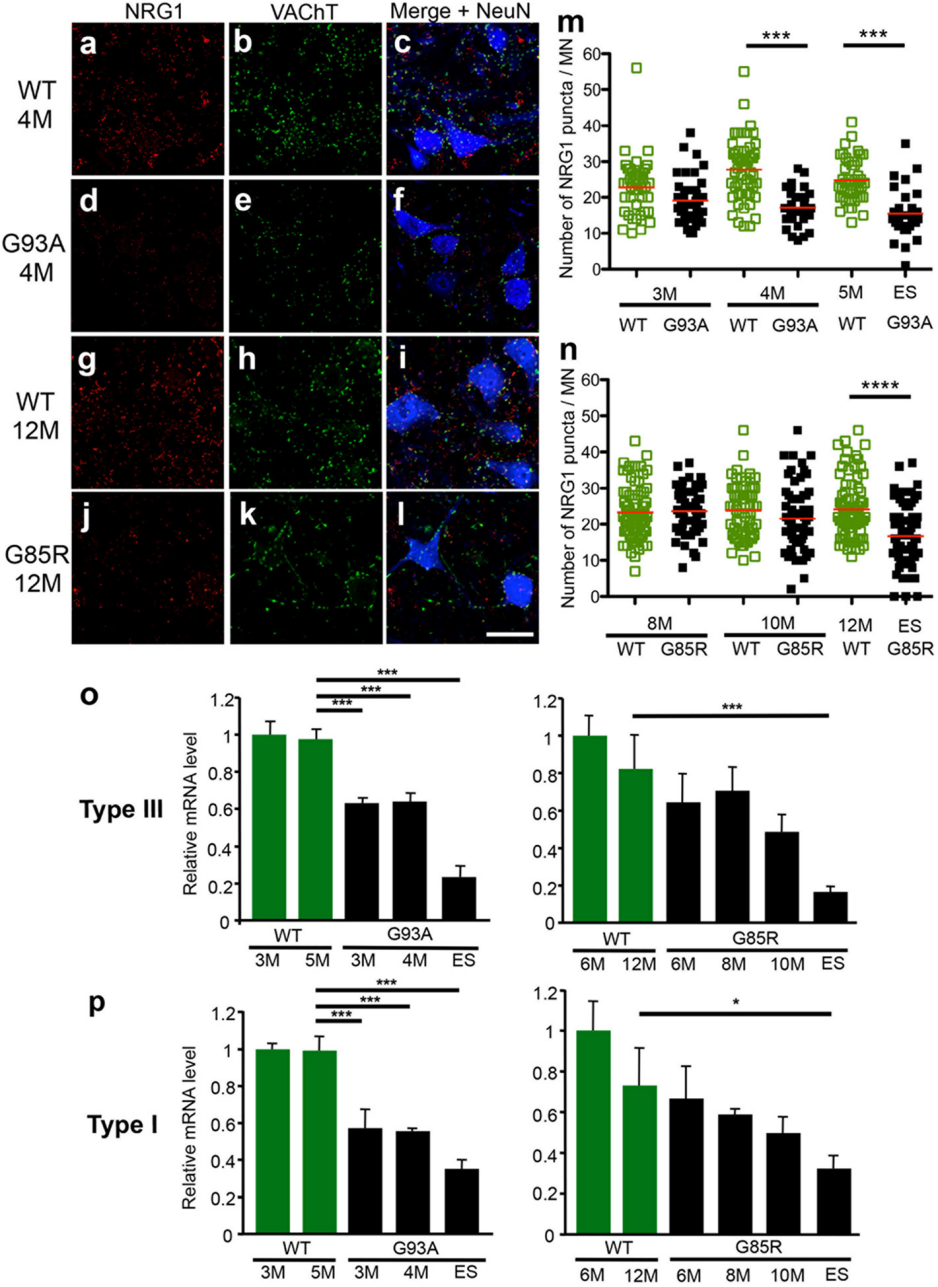
Age-dependent losses of NRG1 in the lumbar spinal cord of SOD1^G93A^ and SOD1^G85R^ mice. (**a**–**l**) Representative images of the lumbar spinal cord of 4 month old wild-type (WT) mice (**a**–**c**), SOD1^G93A^ (G93A) mice (**d**–**f**), 12 month old wild-type (WT) mice (**g**–**i**), and SOD1^G85R^ (G85R) mice (**j**–**l**) stained with the antibodies for NRG1 (**a**, **d**, **g**, **j**), VAChT (**b**, **e**, **h**, **k**), and NRG1/VAChT/NeuN (**c**, **f**, **i**, **l**). Bar: 50 μm (**m**, **n**) Quantification of the number of NRG1-positive puncta per motor neuron in wild-type and SOD1^G93A^ mice (**m**) or wild-type and SOD1^G85R^ mice (**n**) at indicated ages. ES denotes endstage. At least 30 (SOD1^G93A^) or 60 (SOD1^G85R^) motor neurons from mutant SOD1 (*n* = 3−5) or wild-type mice (*n* = 2−3) were analyzed for NRG1-positive puncta. Fewer number of motor neurons were analyzed in symptomatic SOD1^G93A^ mice due to loss of well-defined shaped motor neurons. *Red bars* represent average from both groups. ***: *p* < 0.001, ****: *p* < 0.0001 (Dunn’s Multiple Comparison test (**m**) or Bonferroni’s Multiple Comparison test (**n**)). (**o**, **p**) mRNA levels of NRG1 type III (**o**) and type I (**p**) in the lumbar spinal cords of wild-type, SOD1^G93A^, and SOD1^G85R^ mice at indicated ages. The mean mRNA levels of NRG1 isoforms relative to the ones of wild-type mice at 3 month old (*left*) or 6 month old (*right*) obtained by quantitative PCR analysis were plotted. *: *p* <0.05, ***: *p* <0.001 (Tukey’s multiple comparison test). Error bars denote SD

Several studies have investigated the number and morphology of C-boutons synapsing onto spinal motor neurons of SOD1-ALS mice by using VAChT immunoreactivities or ultrastructural analysis, revealing that C-boutons are mostly preserved until the symptomatic phase [10–12]. To examine whether NRG1 expression is affected in SOD1-ALS mice, we analyzed NRG1-positive puncta in lumbar motor neurons of SOD1^G93A^ and SOD1^G85R^ mice, the rodent models widely used for inherited ALS (Fig. 2a–l). In both mutant SOD1 mice, the numbers of NRG1-positive puncta were decreased at symptomatic stages compared with age-matched, non-transgenic littermates (Fig. 2m, n). The numbers of VAChT-immunopositive puncta, which reflect the number of C-boutons, were also decreased in the symptomatic SOD1^G93A^ and SOD1^G85R^ mice (Additional file 1: Fig. S1). The mRNA levels for type I and type III NRG1 were also decreased in the lumbar spinal cords of symptomatic SOD1^G93A^ and SOD1^G85R^ mice in an age-dependent manner (Fig. 2o, p). These results indicate that both NRG1 transcript and protein levels were decreased in the lumbar spinal cords of symptomatic SOD1^G85R^ and SOD1^G93A^ mice.

### Loss of NRG1-positive puncta in the spinal motor neurons of sporadic ALS patients

**Fig. 3:**
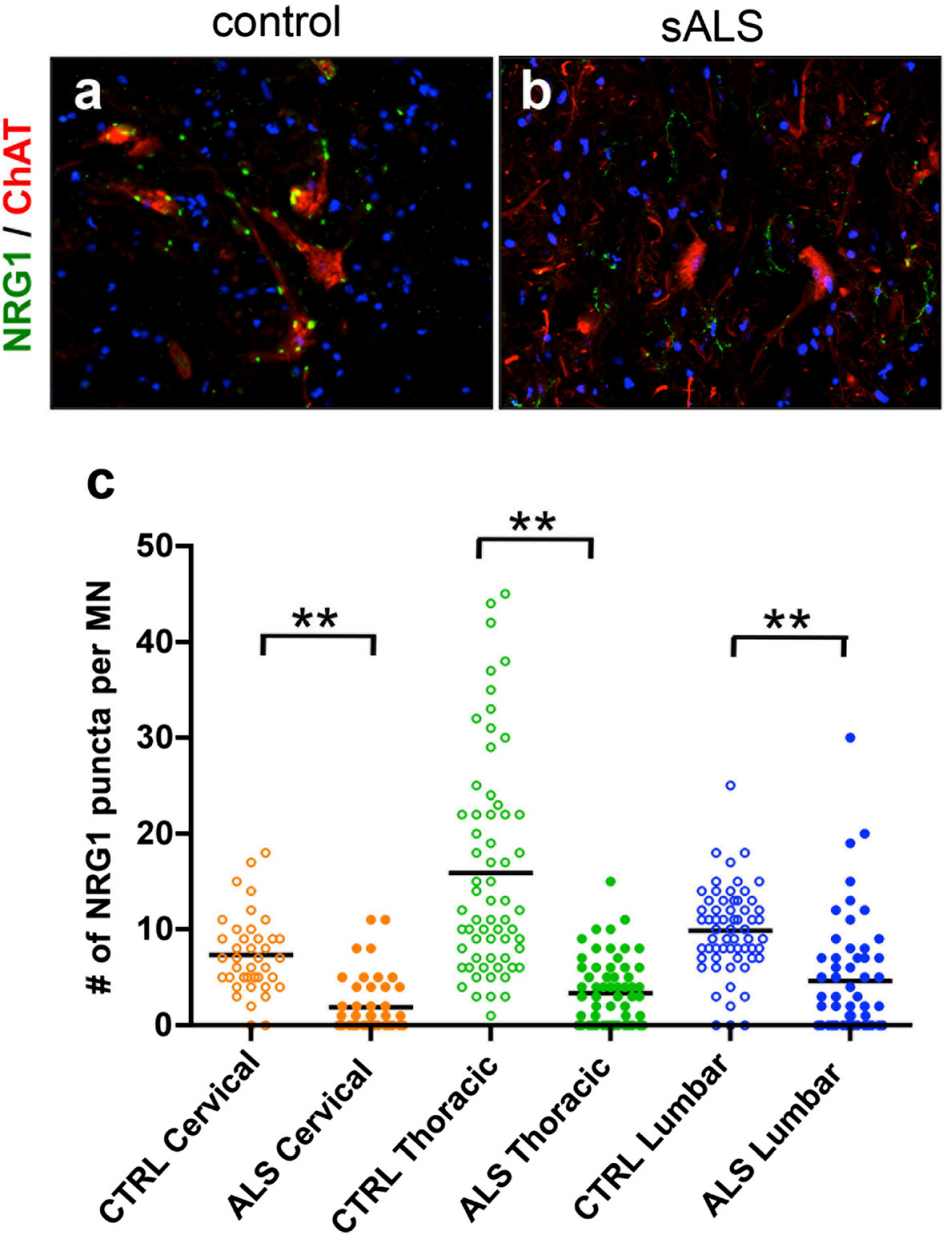
Loss of NRG1-positive puncta in the spinal motor neurons of sporadic ALS and control patients. (**a**, **b**) Representative images of lumbar spinal cord of the control (**a**) and sporadic ALS patients (**b**) stained with NRG1 (*green*), ChAT (*red*), and DAPI (*blue*). Magnification: 20x. (**c**) Quantification of mean NRG1-positive puncta per motor neuron from indicated regions of spinal cords from control and ALS patients (*n* = 3, each). **: *p* <0.01 by one-way ANOVA

Previous reports demonstrated a decreased number of C-boutons in postmortem spinal motor neurons in sporadic ALS [8]. However, it is not established whether NRG1 localization and loss of its expression follow a similar pattern in sporadic ALS to that of ALS mice. We found that NRG1 was localized to the surface of motor neuron somata and proximal processes in human spinal cord (Fig. 3a), consistent with its localization in mice. Furthermore, NRG1 puncta surrounding motor neurons were substantially lost in sporadic ALS (Fig. 3b). Examination of cervical, thoracic, and lumbar spinal cord tissues from three sporadic ALS and control patients revealed that the average number of NRG1 puncta per motor neuron was significantly reduced in the spinal motor neurons of sporadic ALS at all spinal levels (Fig. 3c).

### Viral-mediated delivery of type III NRG1 extends the survival time of SOD1^G93A^ mice

**Fig. 4:**
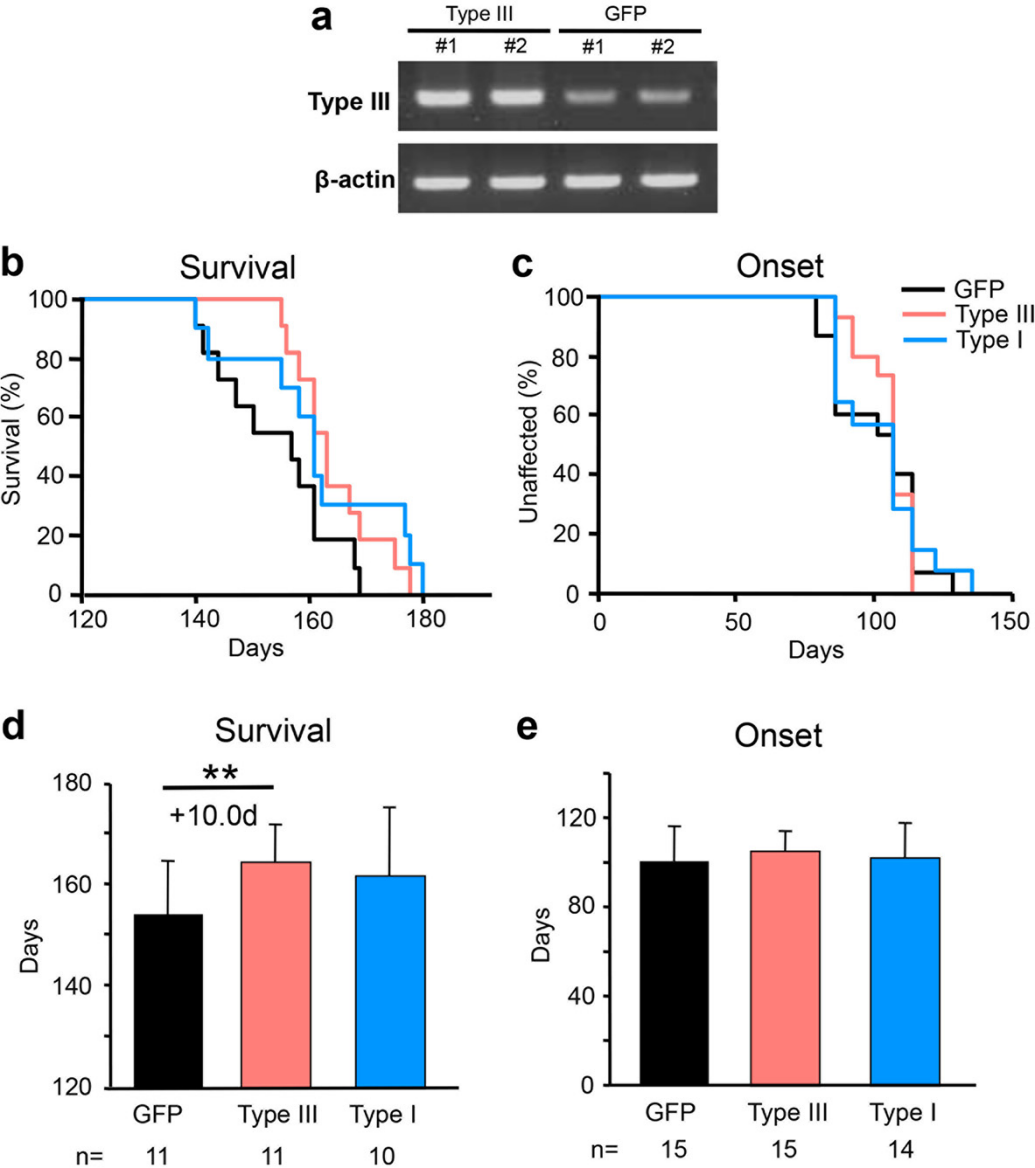
Virus-mediated delivery of NRG1 extends the survival time of SOD1^G93A^ mice. (**a**) Semi-quantitative reverse transcription PCR for Type III-NRG1 and β-actin mRNA in AAV-Type III-NRG1 injected- and AAV-GFP injected- spinal cords. This PCR reaction amplifies endogenous NRG1 as well (GFP). (**b**, **c**) Kaplan-Meier curve of onset (**c**) and survival time (**b**) for SOD1^G93A^ mice injected with AAV-Type III-NRG1 (*red*) (survival time: male = 3, female = 8, onset: male = 5, female = 10), AAV-Type I-NRG1 (*blue*) (survival time: male = 4, female = 6, onset: male = 6, female = 8), or AAV-GFP (black) (survival time: male = 4, female = 7, onset: male = 6, female = 9). (**d**, **e**) Mean survival (**d**) and onset time (**e**) of SOD1^G93A^ mice injected with indicated virus are plotted. Error bars denote SD. **: *p* < 0.05 (Gehan-Breslow-Wilcoxon test)

Loss of NRG1 puncta at C-boutons of motor neurons in both sporadic ALS patients and two distinct lines of SOD1-ALS mice prompted us to test whether NRG1 has a neuroprotective potential for motor neuron disease. Adeno-associated virus (AAV) encoding full-length type III and type I mouse NRG1 were administered in lumbar spinal cords of SOD1^G93A^ mice (Fig. 1a). Overexpression of NRG1 by AAV-NRG1 was confirmed by elevated levels of NRG1 transcripts when compared with control injection of AAV-GFP (Fig. 4a). Presymptomatic administration of AAV-Type III-NRG1 did not alter the time of disease onset (Fig. 4c, e), however, significantly extended the survival time of SOD1^G93A^ mice (AAV-Type III-NRG1: 164.2 ± 7.4 days; AAV-GFP: 154.2 ± 10.4 days; Fig. 4b, d). Administration of Type I-NRG1 also showed tendency to extend survival time, however, it did not reach statistical significance (AAV-Type I-NRG1: 161.4 ± 14.0 days; Fig. 4b–e). Symptomatic treatment with AAV-NRG1 had a marginal effect on extending survival time (AAV-Type I-NRG1: 165.3 ± 13.1 days; AAV-Type-III-NRG1: 162.5 ± 12.5 days; AAV-GFP: 159.5 ± 11.8 days; Additional file 1: Fig. S2). These results suggest a neuroprotective role of NRG1 in motor neuron degeneration.

### Viral-mediated delivery of NRG1 restores the number of NRG1-positive puncta and increases the size of motor neuron somata in SOD1^G93A^ mice

**Fig. 5:**
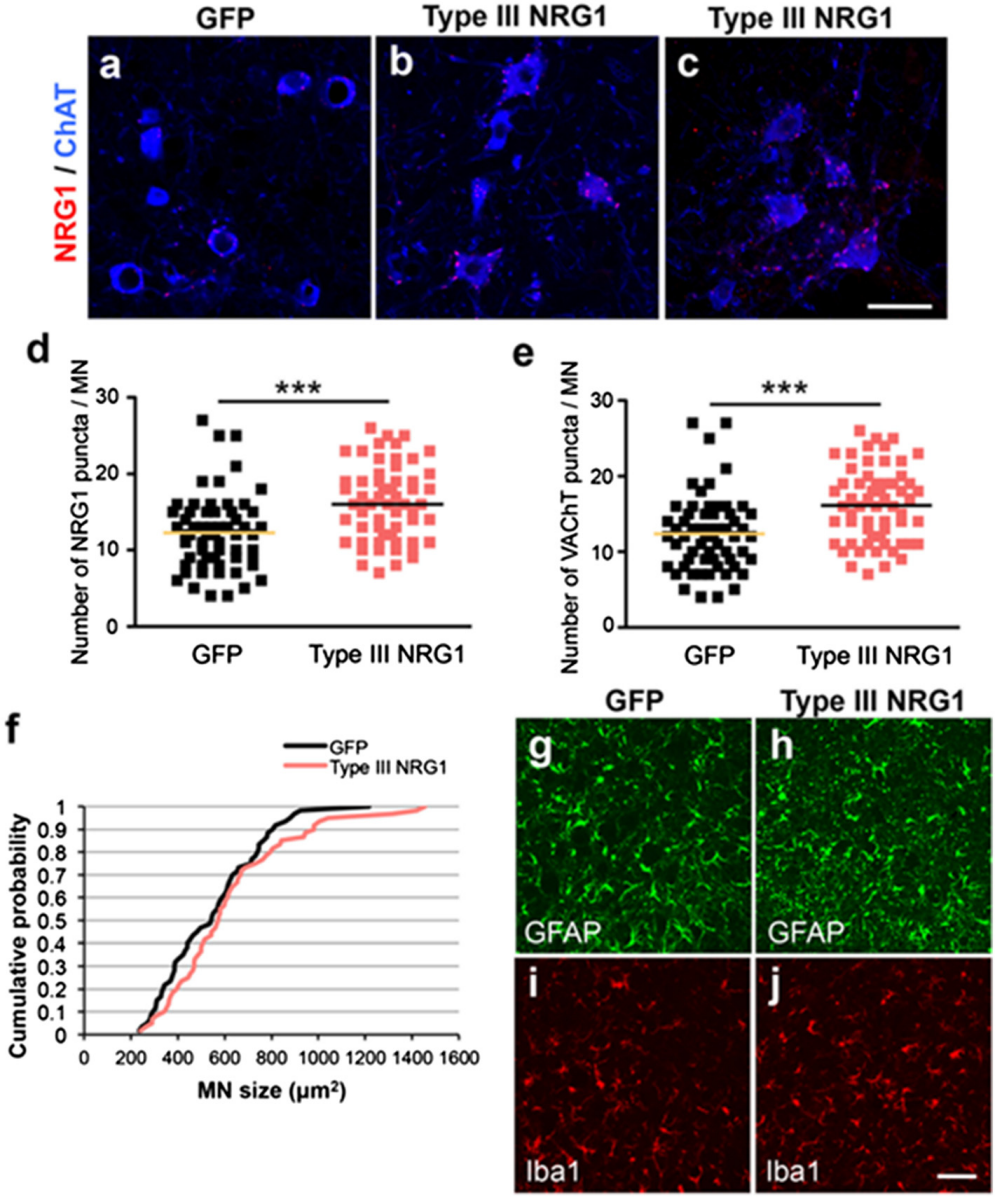
Virus-mediated delivery of Type III-NRG1 restores the number of NRG1-positive puncta and increases the size of motor neuron soma in SOD1^G93A^ mice. (**a**–**c**) Representative images for the lumbar motor neurons of SOD1^G93A^ mice injected GFP (**a**) or Type III-NRG1 (**b**, **c**) encoding AAV. The lumbar spinal cord sections were stained with antibodies for NRG1 (*red*) and ChAT (*blue*). Bar: 50 μm. (**d**, **e**) Quantification for the average number of NRG1-positive puncta (**d**) or VAChT-positive C-boutons (**e**) per motor neuron soma. Sixty motor neurons from GFP- or Type III-NRG1-injected symptomatic SOD1^G93A^ mice (*n* = 3 each) were analyzed for NRG1-positive puncta. The bars indicate averages for both groups. ***: *p* < 0.001, unpaired *t*-test. (**f**) Cumulative probability plot for the size of motor neuron soma. Sixty VAChT-positive lumbar motor neurons from SOD1^G93A^ injected with AAV-GFP (*black*) or AAV-Type III-NRG1 (*red*) were analyzed for the surface area of soma (*n* = 3 per each group). Non-statistical significance among two groups, Kolmogorov-Smirnov test. (**g**–**j**) Representative images for the lumbar spinal cord sections of SOD1^G93A^ mice injected with AAV-GFP (**g**, **i**) or AAV-Type III-NRG1 (**h**, **j**) stained with antibodies for GFAP (**g**, **h**) or Iba1 (**i**, **j**). Bar: 50 μm

To uncover the neuroprotective mechanism of NRG1 in SOD1^G93A^ mice, we quantified the number of NRG1-positive puncta surrounding lumbar motor neurons. In comparison with GFP-transduced animals, Type III-NRG1 administration restored the number of NRG1-immunoreactive puncta as well as C-boutons immunoreactive for VAChT in lumbar motor neurons (Fig. 5a–e). In addition, the size of remaining motor neurons showed a trend toward enlargement in Type III-NRG1-trasnduced mice compared to GFP-transduced mice (Fig. 5f). Activation of astrocytes and microglia was evaluated by immunostaining for glial fibrillary acidic protein (GFAP) and Iba1, respectively, revealing no significant difference in the degree of glial activation in endstage SOD1^G93A^ mice treated with Type III-NRG1 or GFP (Fig. 5g–j). Marginal changes in glial cells in SOD1^G93A^ mice overexpressing Type III-NRG1 may be attributed to the predominant expression of exogenous NRG1 in the neurons (Additional file 1: Fig. S3).

### ErbB4 and ErbB3 expressions are reduced in SOD1^G93A^ motor neurons

**Fig. 6:**
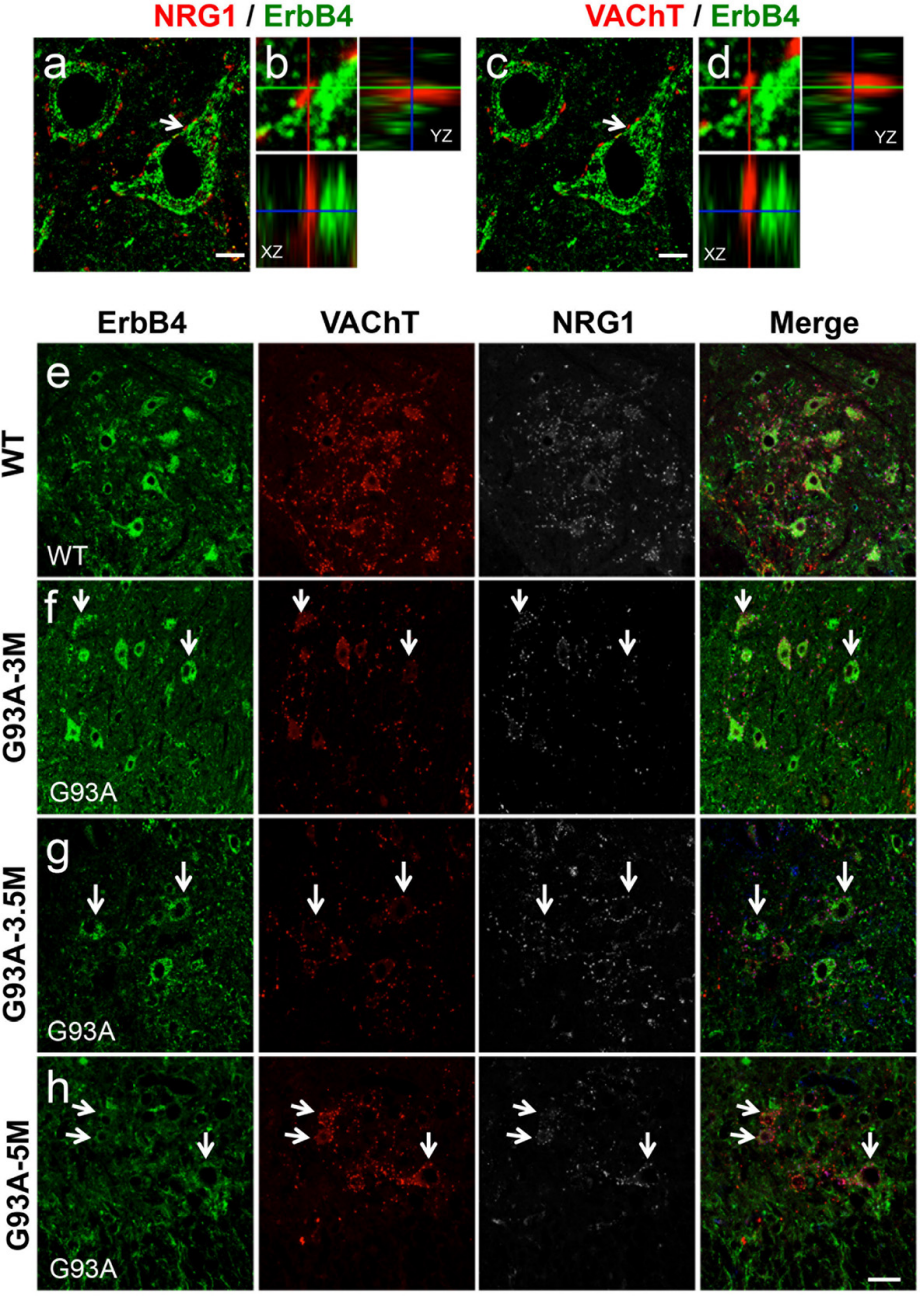
Reduction of ErbB4 expression precedes loss of NRG1 puncta in the lumbar motor neurons of SOD1^G93A^ mice. (**a**–**d**) The lumbar spinal cord sections from 5 months old wild-type mice were stained with the antibodies for ErbB4, VAChT, and NRG1. The representative images for motor neurons stained with NRG1/ErbB4 (**a**) or VAChT/ErbB4 (**c**) with corresponding magnified Z axis-images of the C-bouton (**b**, **d**, *arrows*). Bars: 10 μm. (**e**–**h**) The lumbar spinal cord sections from 5 months old wild-type (WT; **e**) or SOD1^G93A^ mice at 3 (**f**), 3.5 (**g**) and 5 months old (**h**) were stained with the antibodies for ErbB4, VAChT, and NRG1. Representative images in the anterior horn area were shown with the merged images. *Arrows* indicate the motor neurons surrounded by VAChT- and NRG1-positive puncta showed diminished expressions of ErbB4. Bars: 50 μm

**Fig. 7:**
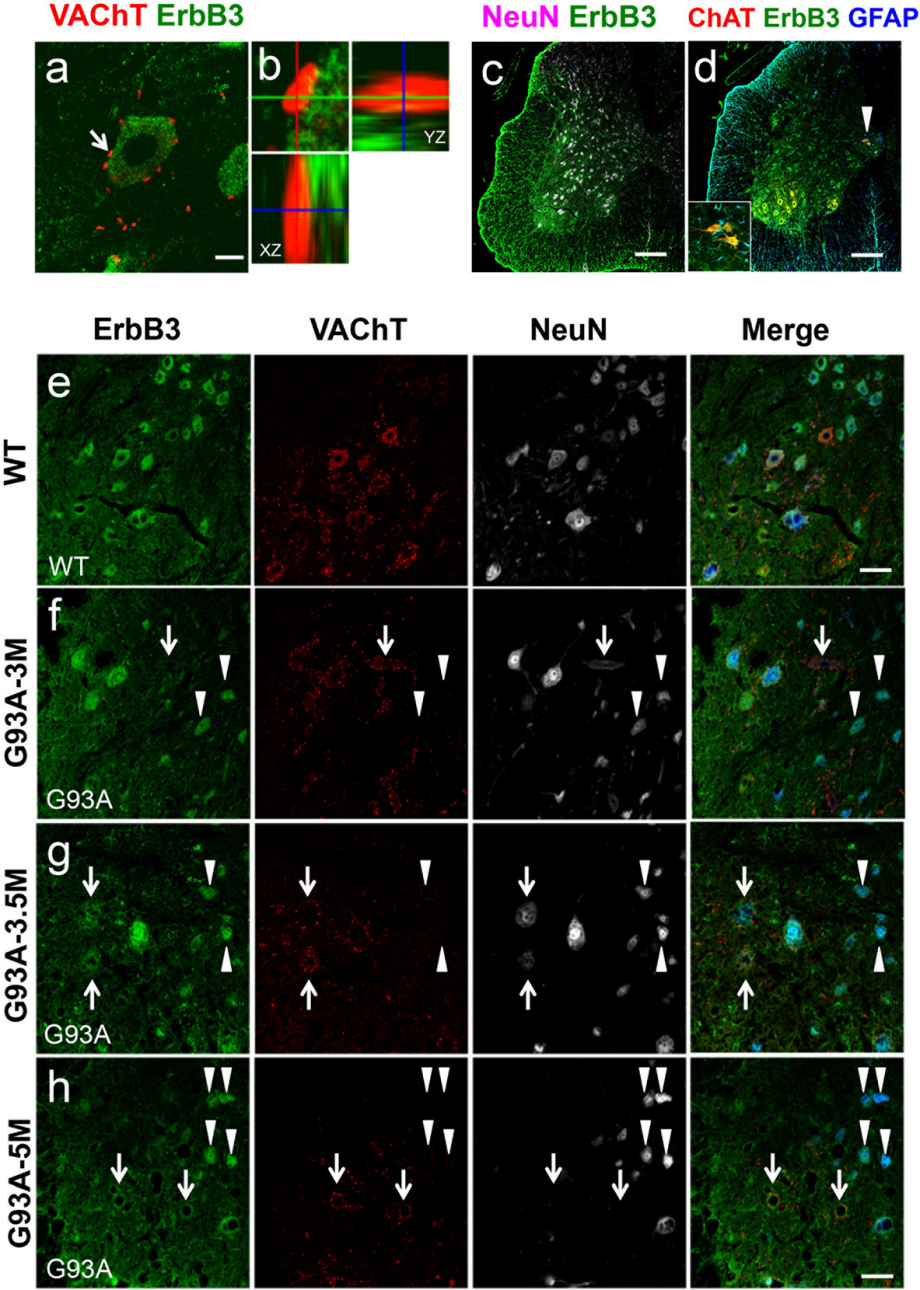
Reduction of ErbB3 expression in the lumbar motor neurons of SOD1^G93A^ mice. (**a**–**d**) The lumbar spinal cord sections from 5 months old wild-type mice were stained with the antibodies for ErbB3, VAChT, GFAP, and NeuN. The representative images for motor neurons stained with VAChT/ErbB3 (**a**) with corresponding magnified Z axis-images of the C-bouton (**b**, *arrow*). Bar: 10 μm. (**c**, **d**) Images for the lumbar spinal cord sections from 5 months old wild-type mice stained with the antibodies for ErbB3 and NeuN (**c**), or ErbB3, ChAT, and GFAP (**d**). *Arrowhead* and inset indicate ErbB3 expressions in ChAT-positive partition cells and their magnified image, respectively. Bars: 200 μm (**c**, **d**). (**e**–**h**) The lumbar spinal cord sections from 5 months old wild-type (WT; **e**) or SOD1^G93A^ mice at 3 (**f**), 3.5 (**g**) and 5 months old (**h**) were stained with the antibodies for ErbB3, VAChT, and NeuN. Representative images in the anterior horn area were shown with the merged images. *Arrows* indicate the motor neurons surrounded by VAChT-positive puncta showed diminished levels of ErbB3. *Arrowheads* show the VAChT-negative, non-motor neurons with preserved ErbB3 expressions in SOD1^G93A^ mice. Bar: 50 μm (**e**–**h**)

Dominant mutations in the gene for ErbB4, encoding a receptor for NRG1, is causative for inherited ALS (ALS19) [19]. Considering that NRG1 expression in SOD1-ALS models is linked to neurodegeneration, it is of interest to examine whether ErbB4 is dysregulated in SOD1-ALS models. In spinal cords of wild-type mice, we observed punctate ErbB4 staining on large spinal neurons surrounded by VAChT-immunopositive puncta, indicating that ErbB4 is enriched in motor neurons (Fig. 6a, c, e). Double immunofluorescence studies revealed that ErbB4 was co-localized neither with NRG1 nor VAChT (Fig. 6a–d). These results indicate that ErbB4 is not localized in C-boutons but is predominantly expressed in the membrane compartment of spinal motor neurons. Compared with wild-type mice (Fig. 6e), immunoreactivity of ErbB4 is decreased in some motor neurons of SOD1^G93A^ mice around the time of disease onset (3–3.5 months old, Fig. 6f, g), followed by more significant losses of ErbB4 in the remaining SOD1^G93A^ motor neurons at symptomatic stages (Fig. 6h). Intriguingly, we observed a partial preservation of NRG1/VAChT puncta in the SOD1^G93A^ motor neurons with diminished ErbB4 expressions (Fig. 6f–h, arrows). We also examined the expression of ErbB3, another receptor for NRG1. ErbB3 is known to be predominantly expressed in oligodendrocytes, Schwann cells, and dorsal root ganglia [24]. However, our staining revealed that ErbB3 also expressed in the somata of neurons including motor neurons in the adult mouse spinal cord as previously reported in rat (Fig. 7a–e) [25]. In addition, ErbB3 also expressed in astrocytes (Fig. 7d), and partition cells (Fig. 7d, arrowhead and inset, Additional file 1: Fig. S4). Although ErbB3 expressed in almost all spinal neurons, it was not co-localized with VAChT (Fig. 7a, b). As like ErbB4, ErbB3 expression was reduced in some motor neurons of SOD1^G93A^ mice around the time of disease onset (Fig. 7f, g), and diminished further toward the symptomatic stage (Fig. 7h). These data suggest that the losses of ErbB4 and ErbB3 were observed in SOD1^G93A^ motor neurons at disease onset and preceded the decreased expressions of VAChT and NRG1 puncta.

## Discussion

Recently, localization of NRG1 has been documented at cholinergic synapses that innervate spinal motor neurons, called C-boutons [18]. Alterations in the numbers or sizes of C-boutons have been reported in ALS patients and mouse models [8–12, 26, 27]. This, together with the recent discovery that a mutation in ErbB4, a component of NRG1 receptors, is causative for ALS19 [19], suggests that disruption of the NRG1-ErbB4 axis could play a role in ALS neurodegeneration. In this study, we determined that NRG1 is enriched at the post-synaptic face of C-boutons in spinal motor neurons. We also found that expression of NRG1 was significantly lost in symptomatic ALS mice and human ALS, and that expressions of ErbB4 and ErbB3 are reduced in the motor neurons of ALS mice around the disease onset. Finally, we report that viral-mediated delivery of type III NRG1 restored the number of C-boutons and extended survival times of SOD1^G93A^ mice. These results suggest that supplementation of NRG1 is capable of ameliorating motor neuron disease partly through maintenance of C-boutons of spinal motor neurons.

Using immunoreactivity of VAChT, alteration of C-boutons in mutant SOD1 mice reported by the several groups was inconclusive [9–12]. Our analyses revealed moderate loss of C-boutons in two distinct strains of symptomatic mutant SOD1 mice, SOD1^G93A^ and SOD1^G85R^. Discrepancy among these previous studies may be attributed to a difference in methods and choice of motor neurons for quantification. Since moderate losses of C-boutons and NRG1 expression in motor neurons were observed almost contemporaneously, reduction of NRG1 in motor neurons may occur not through dysfunction of C-bouton mediated cholinergic inputs but through the general impairment of pre-degenerating motor neurons. In addition, although Song and colleague reported that type I NRG1 mRNA was elevated in end-stage SOD1^G93A^ mice [20], we observed the levels of both type I and type III NRG1 mRNAs were reduced in the spinal cord of symptomatic SOD1^G93A^ and SOD1^G85R^ mice. Discrepancy between two studies might be partly attributed to the genetic background of SOD1^G93A^ mice used in the study by Song and colleague. Future studies will reveal the detailed expression profiles of NRG1 splice variants in ALS mice.

Our detailed immunofluorescence study revealed that NRG1 immunoreactivity co-localized with the post-synaptic proteins, m2 muscarinic receptors and voltage-gated Kv2.1 channels, and not with the pre-synaptic protein, VAChT. These findings indicated that NRG1 was localized in the post-synaptic face of C-boutons, a finding similar to the one reported by Gallart-Palau et al [9]. This indicates that the source of NRG1 expression is motor neurons themselves. In the nervous system, NRG1 plays multiple roles in neural development, synaptic plasticity and transmission, Schwann cell differentiation, and regulation of myelin sheath thickness [16, 17]. Although one group reported ErbB4 receptor is localized in presynaptic terminal of C-boutons [9], our staining revealed that ErbB4 is not co-localized with VAChT. Instead, ErbB4 immunoreactivity is mainly observed in motor neuron somata with punctate staining. Moreover, we observed for the first time that ErbB4 and ErbB3 expressions in motor neurons decreased just prior to the losses of NRG1/VAChT puncta in mutant SOD1 mice, despite that the mechanism of progressive loss of ErbB4/ErbB3 remains unclear. Progressive losses of ErbB4/B3 in the motor neurons may explain that neuroprotection was achieved by the administration of type III-NRG1 at the presymptomatic stage, not at the symptomatic stage.

Previous studies showed that the number of C-boutons per motor neuron was transiently increased in P8-P30 SOD1^G93A^ mice [12, 27] and that C-boutons become enlarged in response to motor neuron damage, leading to increased excitability of motor neurons [12, 26]. These phenomena were interpreted as a compensatory mechanism to maintain the function of motor neurons. Our study partially supports this hypothesis, since viral-mediated supplementation of type III-NRG1 provided neuroprotection by increasing the number of C-boutons, rather than the size. On the other hand, our immunostaining revealed that ErbB4 receptor was mainly localized to the motor neuron membrane rather than to partition cells, while ErbB3 expressed in the all spinal neurons including the partition cells. Considering that NRG1 and ErbB4 are selectively localized to the motor neurons in the spinal cord, we propose that NRG1 may provide neuroprotection to motor neurons directly through ErbB4 receptors in motor neuron somata. In many cases, NRG1-ErbB signaling is observed at the synaptic terminal, the interface of nerve axon and Schwann cells, or neuromuscular junction [24]. However, autocrine signaling of NRG1-ErbB is observed in Schwann cell differentiation and remyelination or tumor growth [28–30]. In addition, NRG1 is known to exert neuroprotection through ErbB4 receptor in MPTP models of Parkinson’s disease [31] and cerebral ischemia [32]. Therefore, NRG1 may protect motor neurons through ErbB4 by an autocrine fashion.

Alternatively, it is possible that NRG1 confers neuroprotection indirectly via ErbB3 in the partition cells or their neighboring astrocytes and oligodendrocytes to maintain the number and integrity of C-boutons. Several reports have described the roles of interneurons in SOD1-ALS models. Glycinergic interneurons are degenerated prior to symptomatic onset in SOD1^G93A^ mice, while no significant change in other interneurons, such as GABAergic neurons was found [15]. Although only modest degrees of changes in cholinergic interneurons has been reported, including herein, further examination of the role of interneurons in non-cell autonomous aspects of motor neurodegeneration will provide a more complete picture of pathomechanisms in ALS.

## Conclusions

Our study is the first to demonstrate dysregulation of the NRG1-ErbB4/3 axis in both human and mouse ALS and to provide support for NRG1 supplementation as a possible therapeutic option for motor neuron disease. Considering that NRG1 conferred increased survival in SOD1-ALS mice and its receptors ErbB4/3 were dysregulated, maintenance of the NRG1-ErbB4/3 axis might be crucial for mitigation of SOD1-ALS. Lastly, since NRG1 was also lost in sporadic ALS motor neurons, it may also play a role in motor neuron health in non-SOD1 associated ALS.

## Additional file

Additional file 1:
**Supplementary figures (Figure S1, S2, S3, and S4).** (PDF 2135 kb)

## Abbreviations

AAV: adeno-associated virus
ALS: amyotrophic lateral sclerosis
ChAT: choline acetyltransferase
GFAP: glial fibrillary acidic protein
m2: M2 muscarinic acetylcholine receptor
NRG1: neuregulin-1
PCR: polymerase chain reaction
SOD1: copper/zinc superoxide dismutase-1
VAChT: vesicular acetylcholine transporter
WPRE: woodchuck post-translational regulatory element
