# Supplementary material for: Neuregulin 1 confers neuroprotection in SOD1-linked amyotrophic lateral sclerosis mice via restoration of C-boutons of spinal motor neurons
Source: Acta Neuropathol Commun. 2016 Feb 18;4:15. doi: 10.1186/s40478-016-0286-7 (PMC4758105; doi:10.1186/s40478-016-0286-7)
Supplement: Additional file 1: — Supplementary figures (Figure S1, S2, S3, and S4). (PDF 2135 kb) [file 40478_2016_286_MOESM1_ESM.pdf]

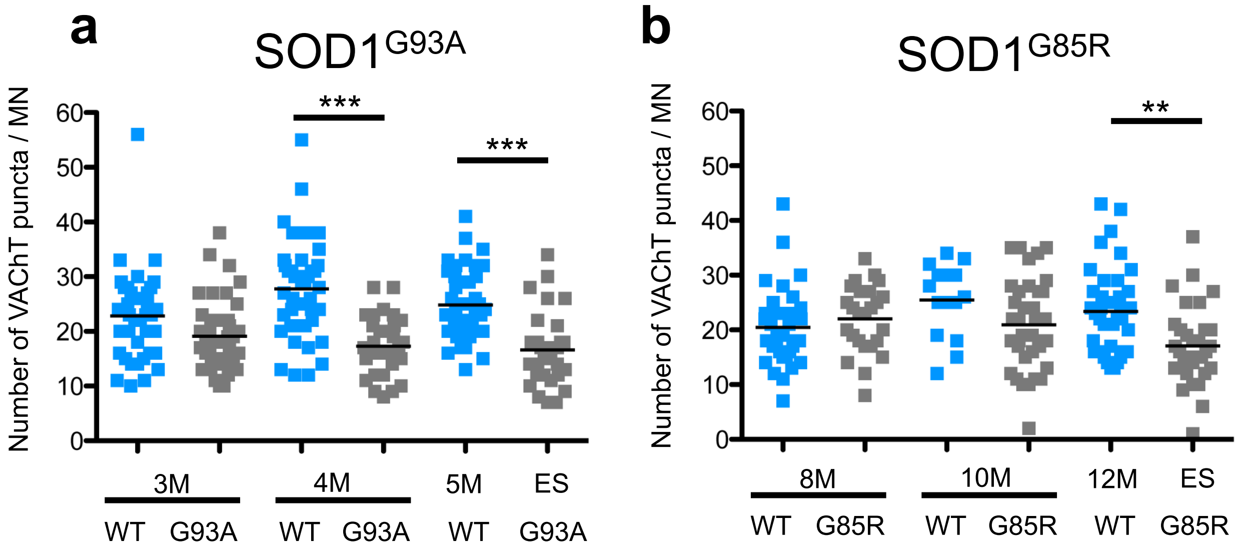

Figure S1. Quantification of VAChT-positive C-boutons in SOD1^{G93A} and SOD1^{G85R} mice.

(a) Numbers of VAChT-immunopositive puncta per motor neuron were quantified in non-transgenic control (WT) and SOD1^{G93A} mice at different time points: 3, 4 and 5 months old. There were significantly fewer number of VAChT-positive puncta in SOD1^{G93A} mice at 4 and 5 months of age. ***: p <0.001, Dunn's Multiple Comparison test. (b) Numbers of VAChT-immunopositive puncta per motor neuron were quantified in non-transgenic (WT) and SOD1^{G85R} mice at different time points: 8, 10, and 12 months old. There were significantly fewer number of VAChT-positive puncta in SOD1^{G85R} mice at 12 months of age. **: p <0.01, Bonferroni's Multiple Comparison test. At least thirty (SOD1^{G93A}) to sixty (SOD1^{G85R}) motor neurons from mutant SOD1 (n=3-5) or wild-type mice (n=2-3) were analyzed for NRG1-positive puncta. Bars represent average from both groups.

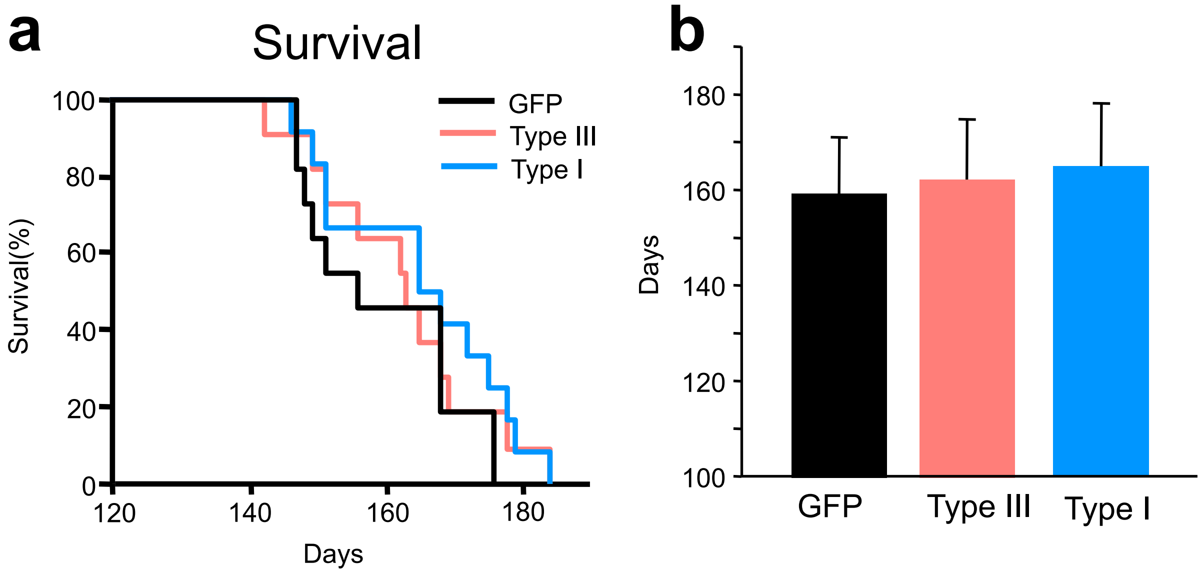

GFP : 159.5 ± 11.8 (days)
Type III: 162.5 ± 12.5
Type I : 165.3 ± 13.1

Figure S2. Survival analysis of SOD1^{G93A} mice injected with AAV-NRG1 after onset of disease.

(a) Kaplan-Meier analysis of survival time of SOD1^{G93A} mice injected with AAV-GFP (black, n=11: male = 4, female = 7), AAV-Type III-NRG1 (pink, n=11: male = 4, female = 7), or AAV-Type I-NRG1 (blue, n=12: male = 4, female = 8) at 105 days old. (b) Mean survival times with standard deviations in (a) are plotted. p = 0.29 (GFP vs Type I), p = 0.53 (GFP vs Type III), Gehan-Breslow-Wilcoxon test.

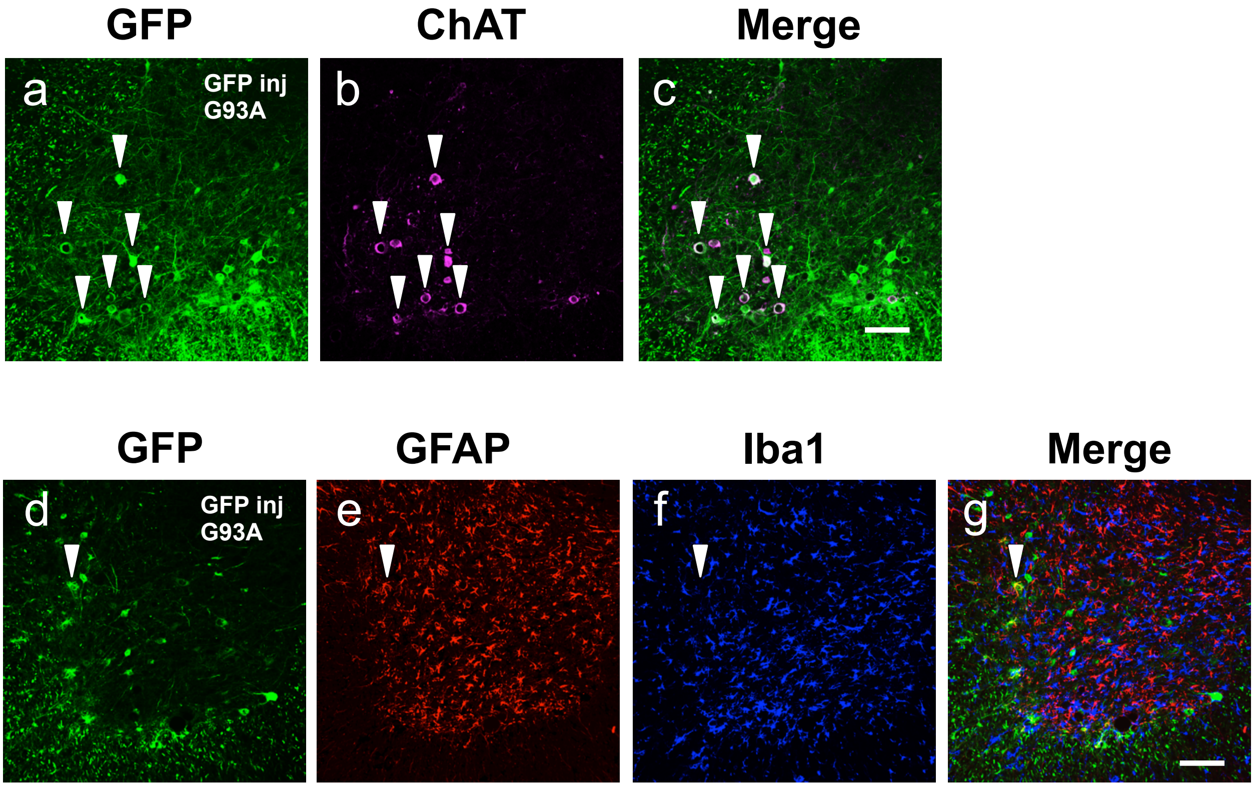

Figure S3. AAV-GFP predominantly expresses in the neurons of lumbar spinal cords of SOD1^{G93A} mice.

(a-g) Representative images for the lumbar spinal cord sections of SOD1^{G93A} mice injected with AAV-GFP. AAV-GFP was visualized without staining (a, d). The lumbar spinal cord sections were stained with the antibodies for ChAT (b), GFAP (e), Iba1 (f), along with the merged images (c, g). (a-c) Arrows indicate the motor neurons immunopositive for GFP and ChAT. (d-g) Arrow indicates GFAP-positive astrocyte expresses AAV-GFP. AAV-GFP expressed predominantly in the neurons including motor neurons, however, very few AAV-GFP signals were detected in glial cells. Bars: 100µm.

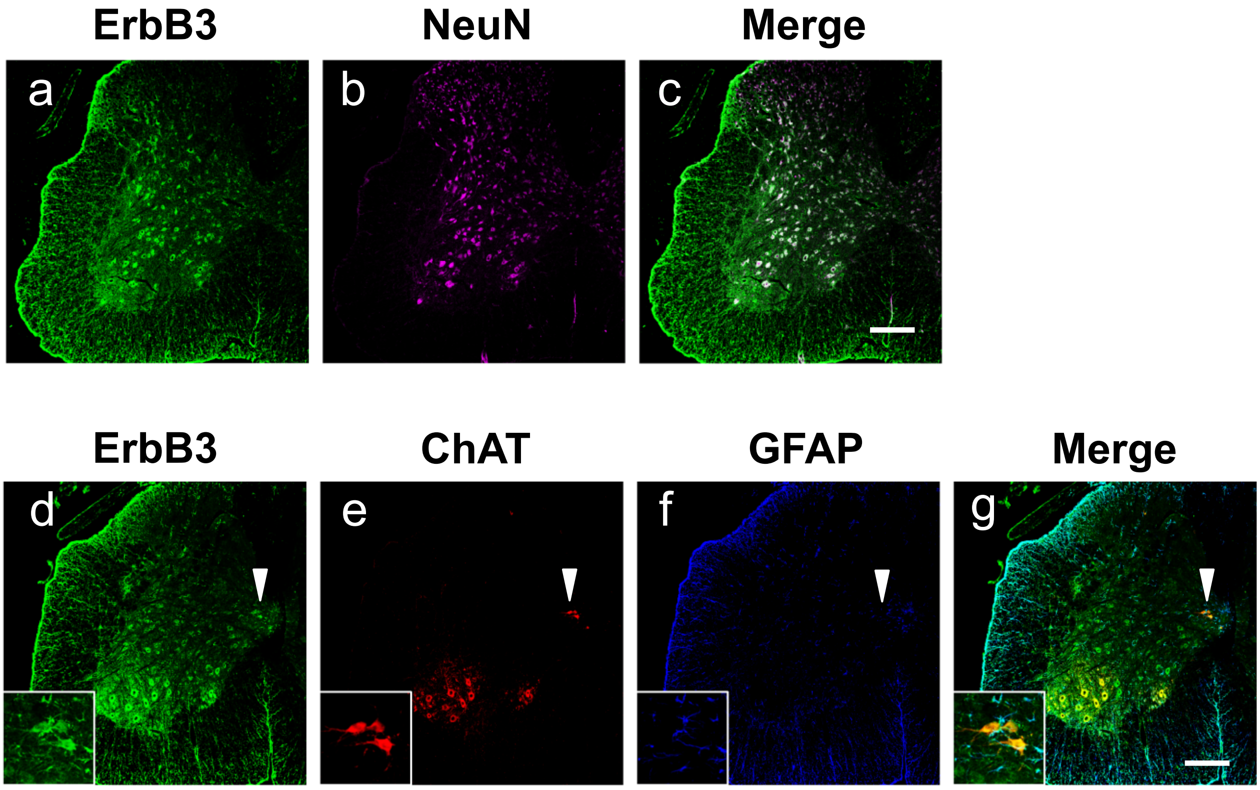

Figure S4. ErbB3 expresses in the spinal neurons including motor neurons and partition cells as well as astrocytes.

(a-c) The lumbar spinal cord sections from 5 months old wild-type mice were stained with the antibodies for ErbB3 (a) and NeuN (b) along with the merged image (c). Merged image is shown in Figure 7c.

(d-g) Images for the lumbar spinal cord sections from 5 months old wild-type mice stained with the antibodies for ErbB3 (d), ChAT (e), and GFAP (f) along with the merged image (g). Arrowhead and inset indicate ErbB3 expressions in ChAT-positive partition cells and their magnified image, respectively. Merged image is shown in Figure 7d. Bars: 200 μm.
